# 

**Published:** 1889-05-04

**Authors:** 


### THE HOSPITAL

A WEEKLY INSTITUTIONAL JOURNAL OF
**Science, Medicine, Nursing & Philanthropy.**

SATURDAY, MAY 4th, 1889.

**The House of Lords and the Hospitals** - - - 63
**Successful Disinfection** - - - - - - 64
**The Doctor's Pulpit.**—Man and his Enemies - - 65
**Within the Wards.**—A Stone in the Kidney - - - 66
**Life in Modern Asylums.**—II. - - - - - - 67
**The Causation of Disease** - - - - - - 68
**Medicine Men of the World.**—II. - - - - - 69
**Notes and News** - - - - - - - - 70
**Everybody's Page** - - - - - - - - 71
**Annotations.**—A Street Ambulance—Religious Intoxication—Shock—Overfeeding - - - - - - - 73
**Turning Over New Leaves.**—Some Recent Fiction—The Characteristics of Genius - - - - - - 74
**"Her Heart's Mistake."** By E. D. Cumming, Author of "An Ordeal by Silence" - - - - - - 75
**A Good Friday Concert at the Edinburgh Castle** 77
**Amusements and Relaxation** - - - - - 78
**Scraps and Gleanings** - - - - - - - 78

**EXTRA SUPPLEMENT—THE NURSING MIRROR.**

**En Passant.**—Combination—Probationers and the Press—Nurse and Dresser—British Nursing Association—A Hint for Midwives—Training at Norwich—Short Items - xv
**The Nursing and Management of the Insane.** By T. Duncan Greenlees, M.B. - - - - - xvi
**Nursing in the Soudan** - - - - - xvi
**Workhouse Infirmary Nursing Association** - xvii
**Everybody's Opinion.**—Recreation for Nurses—Lady Nurses—White Slavery in Hospitals—The Food Question—A Holiday Home - - - - - - - xvii
**Vaccination and its Opponents** - - - - xviii
**Appointments—Vacancies—Notes and Queries** xviii

